# Financial Planning for Retirement: Developing an Integrated Model

**DOI:** 10.1093/geroni/igab046.3285

**Published:** 2021-12-17

**Authors:** Dannii Yeung, Alvin Ho, Alfred Lam

**Affiliations:** 1 City University of Hong Kong, Hong Kong, Hong Kong; 2 City University of Hong Kong, City University of Hong Kong, Hong Kong

## Abstract

With higher life expectancy of the aging population, retirees nowadays will spend a prolonged period of time after retirement. Yet, past studies have consistently revealed a lack of retirement savings among working adults, implying an inadequacy to maintain the quality of life in late adulthood. This study therefore aims to identify the factors influencing the working adults’ intention to purchase financial products for retirement (such as deferred annuity and voluntary contribution to retirement fund) and develop an integrated model of financial planning for retirement. A total of 598 Hong Kong Chinese working adults from diverse age and income groups completed an online survey on intentions to save and purchase specific financial products for retirement. The results of MANCOVA reveal that compared to older workers, younger workers had lower intentions to save and purchase financial products for their retirement even after controlling for their monthly income [F(16,1797)=2.24, p=.003, partial n2=.015]. An integrated model of financial planning for retirement is proposed by incorporating the concepts of the interdisciplinary psycho-motivation model and Theory of Reasoned Actions (χ2 = 40.42, p<.001, CFI = .99, RMSEA = .07, SRMR = .02). In particular, the positive effects of financial literacy, subjective norms, social support, and future time perspective on intention to save and purchase financial products for retirement have been found to be mediated by retirement goal clarity but not attitudes towards retirement. Future direction on promoting younger and older workers’ retirement planning especially in the financial domain will also be discussed in the presentation.

